# The Story of the Insane from Year to Year

**Published:** 1895-10-12

**Authors:** 


### THE STORY OF THE INSANE FROM YEAR TO YEAR.

### ROYAL ASYLUM, MONTROSE.

An increase from 576 to 594 has to be noted. There were 126 admissions, 55 recoveries, and 42 deaths. The percentage of recoveries on admissions stands at 42·64, and that of the deaths at 7·12 on the average number resident. Of the deaths 18 were caused by cerebral diseases and eight by consumption. Dr. Howden's report is a very short one. We learn from it that mechanical restraint has been used with six patients—in three cases for surgical reasons, in one for the excitement of general paralysis, in one for epileptic mania, and in another during maniacal excitement. A block is about to be built for the reception of private patients.

### WARNEFORD ASYLUM, OXFORD.

This hospital contained 86 patients at the close of the year. There were 14 admissions, three recoveries, anl five deaths. The recoveries at the male side stand at 14·3 per cent. of the admissions, and at the female side at 100 per cent. This shows how valueless such statistics are are unless they embrace long periods, large numbers, and the elimination of every source of fallacy. Intemperance in drink does not figure in the year's admissions as a cause of the insanity, and hereditary tendency accounts for five of the 14. The average weekly cost per head amounts to 27s. 8d., and we are again pleased to record that much charitable work has been carried out during the year. There were three patients who paid 5s. a-week, two who paid 8s., 17 who paid 10s., 15 who paid 15s., and three who paid £1. Fourteen patients who were in residence for less than a year paid sums varying from 5s. to 25s. a-week, so we may conclude that this hospital does really relieve those for whom it was originally intended. Patients able to pay for their maintenance pay £2 2s. a-week, but epileptics and paralytics are not admitted.

### MORPETH ASYLUM, NORTHUMBERLAND.

Here there was during last year a slight decrease in the numbers; 603 patients were in residence on January 1st, and 599 on December 31st. There were 156 admissions, 63 recoveries, and 54 deaths. Calculated on the admissions the recoveries stand at 40·9 per cent., and the deaths are 9 per cent. calculated on the average number resident. Cerebral and spinal diseases account for 26 of the deaths, consumption for 15, and pneumonic phthisis for two. Of the year's admissions 12 were driven insane by domestic trouble and adverse circumstances, 39 by intemperance in drink, 33 by hereditary influences, 36 by "previous attacks," and in 38 the cause was unknown. The asylum is again full, and Dr. McDowall thinks "the time has arrived for this committee and the County Council of Northumberland to take into consideration the provision of additional accommodation for male pauper lunatics." A contract has been entered into with the Menston Asylum for the reception of 20 patients, of course, at a much higher rate than they could be maintained in Morpeth. In turning over the leaves of county asylum reports, it is curious to notice how often committees of visitors neglect this part of their duties, and instead of building a few years before the accommodation is actually needed, postpone building operations from time to time, until in some cases the extra money expended on boarding out would have been a material help in erecting the additions. We are pleased to notice that Jane Shaw, the head laundress, and Bridget Monaghan, a nurse, have been each granted a superannuation allowance of £26 per annum. The weekly charge for maintenance is 9s. 7½d.

### CORK DISTRICT ASYLUM.

The limits of accommodation are given at 1,199 beds, and on January 1st the asylum contained 1,123 patients. On the last day of the year the numbers had risen to 1,221. There were 323 admissions, 111 recoveries, 25 discharged relieved, and 76 deaths. The recoveries stand at 34·3 per cent. of the admissions, and the deaths at 6·4 per cent. on the average number resident. Nine of the deaths were from cerebral or spinal diseases, general paralysis being the cause in only one case, and 31 were from consumption. So-called "moral causes" are stated to have caused the insanity in 29 of the year's admissions, intemperance in drink accounts for 22, and hereditary influences for the same number. "Previous attacks" are put down for 69 cases. There was a slight outbreak of enteric fever, and Dr. Woods says he is "altogether at a loss to find a cause for the two male patients being attacked as both had been living in the new building for some weeks." Like so many English medical superintendents Dr. Woods has been training his nursing staff, and we find that seven of the nurses have passed the examination of the Medico-Psychological Association.

